# Upregulation of Melanogenesis and Tyrosinase Activity: Potential Agents for Vitiligo

**DOI:** 10.3390/molecules22081303

**Published:** 2017-08-04

**Authors:** Chao Niu, Haji A. Aisa

**Affiliations:** 1 Key Laboratory of Plant Resources and Chemistry of Arid Zone, Chinese Academy of Sciences, Urumqi 830011, China; niuchao@ms.xjb.ac.cn; 2 State Key Laboratory Basis of Xinjiang Indigenous Medicinal Plants Resource Utilization, Xinjiang Technical Institute of Physics and Chemistry, Chinese Academy of Sciences, Urumqi 830011, China

**Keywords:** melanogenesis, tyrosinase activity, vitiligo, plant extracts, natural products, synthesized derivatives, analogues

## Abstract

Melanin, the compound primarily responsible in humans for hair, eye and skin pigmentation, is produced by melanocytes through a complicated process called melanogenesis that is catalyzed by tyrosinase and other tyrosinase-related proteins. The abnormal loss of melanin causes dermatological problems such as vitiligo. Hence the regulation of melanogenesis and tyrosinase activity is very important for treating hypopigmentary disorders. Many melanogenesis stimulators have been discovered during the past decade. This article reviews recent advances in research on extracts and active ingredients of plants, synthesized compounds with stimulating effect on melanin synthesis and tyrosinase activity, as well as their influence on the expression of related proteins and possible signaling pathways for the design and development of novel anti-vitiligo agents.

## 1. Introduction

**Figure 1:**
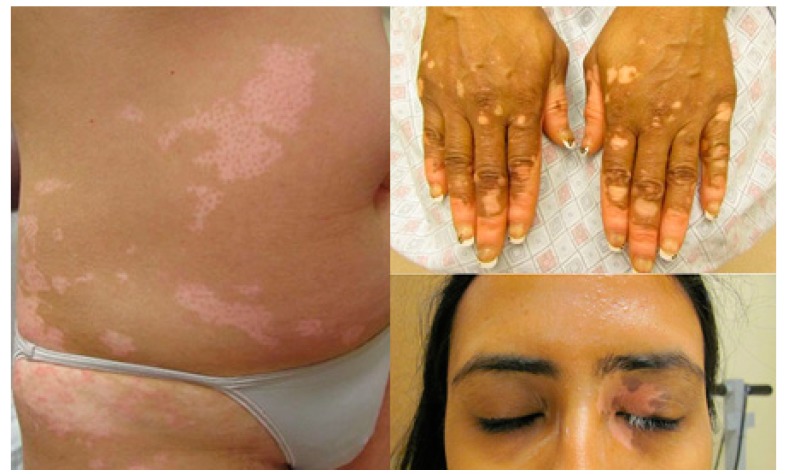
Vitiligo with typical lesions of the face and trunk [3].

Vitiligo is an acquired chronic depigmentation disorder of the skin resulting from selective destruction of melanocytes. Celsus was the first to use the term vitiligo in his Latin medical classic De Medicina during the second century B.C [1,2]. It is clinically characterised by the development of white macules (Figure 1) [3] due to the loss of functioning melanocytes in the skin or hair, or both [4]. The prevalence of the disease is often referred to as 0.05–1% of the world’s population and it is the most frequent cause of depigmentation worldwide [5]. Although essentially asymptomatic, the psychosocial impact of vitiligo can be devastating, and affected persons are often desperate for an effective therapy. Many possible causes of vitiligo, including immunologic, genetic, stress, neural mechanism, and biochemical factors [6] had been proposed, but the etiopathogenesis of the disease is still enigmatic. However, it is believed that the disease is mainly a result of destruction of melanocytes and obstruction of the melanin synthesis pathway [7,8].

Melanin, derived from dopaquinone, serves a number of valuable physiological functions with the most important being photoprotection of the human skin from ultraviolet (UV) radiation [9]. Melanogenesis takes place in special organelles—the melanosomes in the melanocytes—and can be triggered by a variety of paracrine cytokines including α-melanocyte-stimulating hormone (α-MSH) [10], stem cell factor (SCF) [11], endothelin-1 (ET-1) [12], nitric oxide (NO) [13], adrenocorticotropic hormone (ACTH) [14], prostaglandins [15], thymidine dinucleotide [16] and histamine [17]. These factors all induce melanogenesis through diverse signaling pathways by activating the expression and activation of pigment-related proteins such as microphthalmia-associated transcription factor (MITF), tyrosinase (TYR), tyrosine-related protein-1 (TRP-1) and tyrosine-related protein-2 (TRP-2).

**Figure 2:**
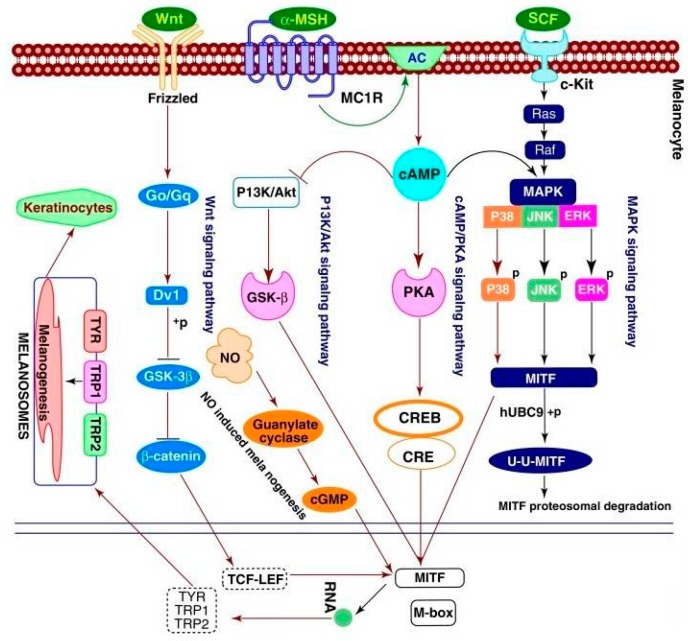
Regulation of melanogenesis through different signaling pathways [18,19].

Figure 2 [18,19] illustrates the most common signaling pathways involved in the synthesis of melanin. All signaling pathways involve MITF, a master regulator of melanogenesis, which upregulates the melanogenesis enzymes TYR, TRP-1 and TRP-2 via binding to the M-box motif in their promoter regions [20]. In addition, MITF regulates melanocyte function including melanocyte differentiation, pigmentation, proliferation and cell survival [21].

**Scheme 1:**
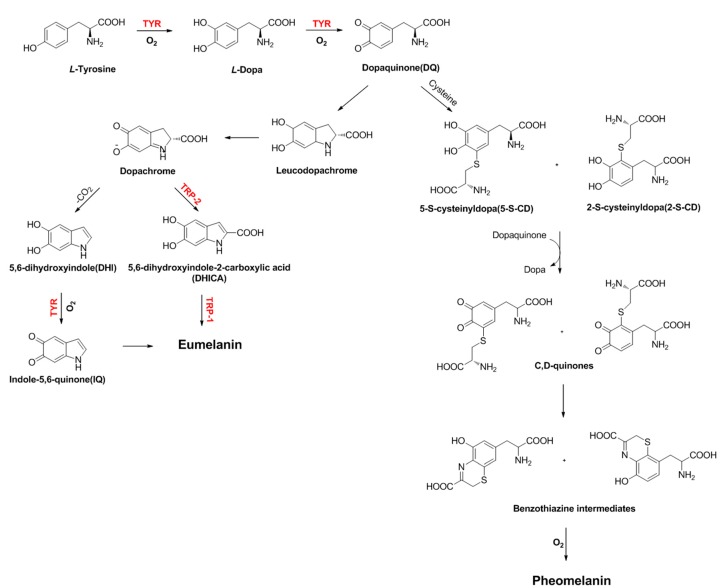
Melanogenesis catalyzed by tyrosinase (TYR), tyrosinase-related protein 1 (TRP-1) and tyrosinase-related protein 2 (TRP-2).

Among them, TYR, TRP-1 and TRP-2 (otherwise called dopachrome tautomerase or DCT) are mainly involved in the transformation of tyrosine into melanin pigments [22]. Melanosomes produce two types of melanin: eumelanin, a brown–black or dark insoluble polymer; and pheomelanin, a red-yellow soluble polymer, formed by the conjugation of cysteine or glutathione [23,24] (Scheme 1). Although three enzymes (TYR, TRP-1 and TRP-2) are involved in the melanogenesis pathway, only TYR is exclusively necessary for melanogenesis.

TYR (EC 1.14.18.1) is a multifunctional membrane-bound type-3 copper protein, which is located in the membrane of the melanosome [25]. TYR is produced only by melanocyte cells. Following its synthesis and consequent processing in the endoplasmic reticulum and Golgi, it is trafficked to melanosomes, wherein the melanin pigment is synthesized. From the structural point of view, two copper ions, each surrounded by three histidines, are responsible for the catalytic activity of TYR [26]. Three different states of the active site have been reported in the pigment formation: oxy-, met- and deoxy-forms. More specifically, at the active site, copper atoms participate directly in hydroxylation of monophenols to diphenols (cresolase activity) and in the oxidation of *o*-diphenols to *o*-quinones (catechol oxidase activity) that enhance melanogenesis [27,28].

TYR is also catalyzing the process of neuromelanin production in which the oxidation of dopamine produces dopaquinones. However, excessive production of dopaquinones results in neuronal damage and cell death [29]. This suggests that tyrosinase might play a significant role in neuromelanin formation in the human brain and responsible for the neurodegeneration associated with Parkinson’s disease and Huntington’s disease [30,31]. Tyrosinase has also been linked to the browning of vegetables and fruits during postharvest and handing process, leading to quick degradation [32,33]. The application of tyrosinase was further extended in the molting process of insects [34].The abnormal accumulation of melanogenesis products may cause cancer (melanoma), age spots, freckle and other dermatological problems [35,36]. The melanogenesis stimulators as skin-pigmenting agents are very important to the occurrence of vitiligo. Thus developing new melanogenesis activators with drug-like properties is very much needed. Here, we focus on the recent discovery of melanogenesis stimulators from all sources, including plant extracts, natural products and laboratory synthetic methods. Moreover, we believe that this perspective will comprise a cumulative source for developing therapeutic agents for inducing repigmentationin vitiligo-affected skin.

## 2. Melanogenesis Stimulators from Different Sources

Many efforts have been made in the discovery and development of melanogenesis stimulators in the last ten years, as it is a very active field of research for academic centres and medical institutions. Some of these stimulators have synthetic origin, but others have been derived from small molecules of natural origin. Worth mentioning is the fact that the plant kingdom has been shown recently to provide a source of chemical structures with promising biological activities. The number of small molecule melanogenesis stimulators is continuously increasing, with most of them being in the early discovery phase. The current stimulators from different sources can be classified into the following two categories:

1. Plant extracts/crude drug extracts (mixtures);
2. Active natural products/synthesized derivatives (single compounds).

### 2.1. Plant Extracts/Crude Drug Extracts (Mixtures)

Traditional herbs and plants are widely used for the skin hypopigmentation in view of their lesser side effects and wealth of sources. The search for new pharmaceuticals for the treatment of vitiligo has increased over the last few years and plant-derived products (e.g., chemical extracts from medicinal plants, herbs, and spices) are becoming increasingly accepted and adopted by the medical industry for this purpose (Table 1).

#### 2.1.1. *Daphne gnidium*

The ethyl-acetate extract of *Daphne gnidium* was recently proved to significantly stimulate production of intracellular and extracellular melanin [37]. Likewise, the tyrosinase activity in B16-F0 cells treated with extraction increased in a time-dependent manner. Later, the effect of chloroform extract of the plant on melanogenesisin murine B16-F0 melanoma was studied as well by the same group [38], and a concentration-dependent stimulation effect on tyrosinase and melanogenesis was observed. It was inferred that the chloroform extract was able to induce differentiation of B16-F0 melanoma cells preventing them from proliferating to the differentiated state. Induction of melanogenesis is considered as a well-known marker of differentiated melanoma cells.

#### 2.1.2. *Moricandia arvensis*

Skandrani et al. [39] evaluated the activity of a chloroform extract of *Moricandia arvensis* on melanogenesis and tyrosinase activity. The results indicated that the chloroform extract significantly promoted production of intra- and extracellular melanin when compared to untreated cells, as well as tyrosinase activity in B16-F0 cells, in a time-dependent manner.

#### 2.1.3. *Ecliptae herba*, *Polygoni multiflori radixpraeparata* and *Rehmanniae radix praeparata*

*Polygoni multiflori radix praeparata* (PMRP), *Ecliptae herba* (EH) and *Rehmanniae radix praeparata* (RRP) are the most frequently-used herbs by traditional Chinese medicine practitioners for the treatment of vitiligo. EH aqueous extract was found to exhibit a synergistic effect on melanocytes by up-regulating tyrosinaseactivity, enhancing melanin synthesis and promoting melanocyte migration as well as elevating MITF protein expression [40]. RRP showed a significant stimulating effect on melanogenesis and MITF protein expression, but no stimulatory effect on tyrosinase activity. In addition, treatment with PMRP and EH promoted the migration of human melanocytes in a type IV collagen-coated transwell migration assay. Theseresults suggest that EH and RRP contain substances with direct enhancing effects on melanogenesis and migration, possibly via their effects on MITF protein expression.

#### 2.1.4. *Cassia alata* and *Cassia occidentalis*

Babitha et al. [41] reported the effect of *Cassia alata* leaf extract on the differentiation, proliferation and migration of melanoblast cells in melb-a melanoblast cells. Result indicated that melanin content increased in a dose-dependent manner. In addition, it induced tyrosinase activity and altered melb-a cell morphology. A transwell migration assay shows that the extract can directly stimulate the migration of melanoblast cells. Similar to the above findings, pod extracts of *Cassia occidentalis*, which is another plant of *Cassia* L., was found to be effective in inducing differentiation and migration ofmouse melanoblast cell line by Babitha and co-workers [42]. Methanolic extract redissolved in DMSO at 12.5 μg/mL was found to cause 3.5 to 3.8-fold melanin induction in melb-a melanoblast cells. Stimulation of tyrosinase activity, dendritogenesis and migration of treated cells were observed as well.

#### 2.1.5. *Pyrostegia venusta*

The flowers of *Pyrostegia venusta* are used in Brazil in the treatment ofwhite patches on the body (such as vitiligo) as a popular folk medicine. It wasdemonstrated that both extracts, leaves and flowers of *Pyrostegia venusta* increased the melanin content in a concentration dependent manner after 4 days of incubation on melanoma cells [43]. Leaves extract promoted enhancement of melanogenesis with a maximum effect of 33.3 ± 3% (3 μg/mL), and the flower extract increased it by 23.4 ± 3% (0.1 μg/mL). However, neither extract was able to cause any change in the tyrosinase activity.

To investigate the effect of this extract in animal models of vitiligo, the hyperpigmentant activities of HE in C56BL/6 mice was also studied later by Moreira et al. [44]. The results showed that extract administered either by gavage (300 mg/kg) ortopically (10%) increased epidermal melanin level (116.5 ± 13% and 100 ± 16.9%, respectively), diminished dermal depigmentation (36.0 ± 6.7% and 38.2 ± 6.2%, respectively), as well as tissue TNF-α levels (68.2 ± 11.6% and 99.2 ± 12.1%, respectively) and cell infiltration (basal levels and 94.3 ± 9.17%, respectively). Only topical treatment with extract altered melanin-specific markers in hair follicles in mice.

#### 2.1.6. *Vernonia* *anthelmintica*

The fruit extract *of Vernonia anthelmintica* is one of the most popular Uyghur medicines used for vitiligo and initially recorded as Kaliziriin ‘*Yao YongZongKu*’ around 300 years ago. The extract was claimed to increase tyrosinase activity and melanin content in a dosage-dependent manner, and the expression of tyrosinase time-dependently in both B16-F10 cells and normal human primary melanocytes as well [45]. Besides, it induced MITF protein expression up-regulation and promoted the level of phosphorylation of p38 mitogen-activated protein kinase (p38-MAPK) markedly at 0–6 h, which illustrated that the extract exerted its improving effect on melanogenesis mainly by p38-MAPK activation and MITF induction of tyrosinase.

Tuerxuntayi et al. [46] of our group reported that Kaliziri increased the tyrosinase activity and melanin content in a dose-dependent manner at concentrations of 5–40 μg/mL, and treatment with 20 μg/mL Kaliziri extract (KZE) enhanced the expression of tyrosinase in B16 melanoma cells in a time-dependent manner.

**Figure 3:**
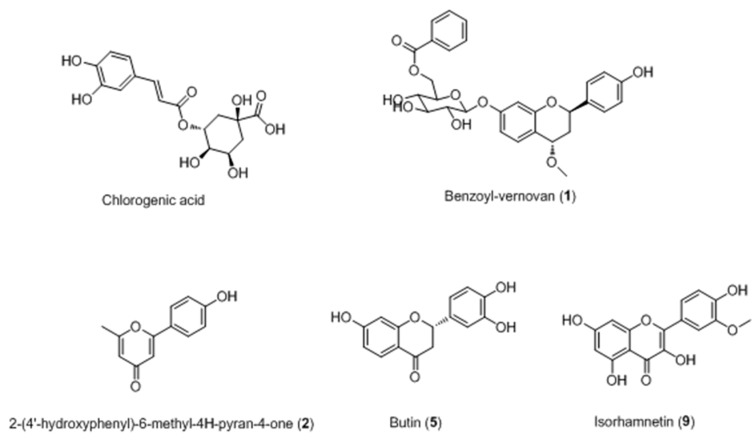
Structures of compounds isolated from *Vernonia anthelmintica* [47,48].

Based on the above research, chlorogenic acid (CGA) [47] and ten compounds (including two novel compounds **1** and **2**) [48] were isolated from the Kaliziri by our group as well (Figure 3). It was speculated that CGA has two side-roles in melanogenesis of B16 melanoma cells as it exhibited different effects on melanogenesis and tyrosinase as the incubation time was extended. CGA is likely a substrate of melanin, but its metabolic products may suppress melanogenesis in B16 melanoma cells by inhibiting tyrosinase activity.

Two new compounds, named benzoyl-vernovan (**1**) and 2-(4′-hydroxyphenyl)-6-methyl-4*H*-pyran-4-one (**2**), together with eight known compounds [2,2′-bis-(3,4-dihydroxy-phenyl]-7,7′-dihydroxy-2,3,2′,3′-tetrahydro-[3,3′]-bichromenyl-4,4′-dione (**3**), 3′,4′,6-trihydroxyaurone (**4**), butin(**5**), butein (**6**), isocarthamin (**7**), luteolin (**8**), isorhamnetin (**9**) and2,4-*trans*-7,4′-dihydroxy-4-methoxyflavan (**10**) were isolated from the KZE (Figure 3). Among them, compounds **5** and **9** were proved to increase melanin content by 2.2% and 30.9% higher than positive control 8-MOP.

#### 2.1.7. *Melissa officinalis*

Pérez-Sánchez et al. [49] reported that extract of *Melissa officinalis* may act as a melanogenic activator since it could promote endogenous melanin production in melanogenesis in a human keratinocytes model. Thirteen major phenolic compounds (including rosmarinic acid, RA) were identified by HPLC-DAD-ESI-IT-MS/MS. Unfortunately, the effects of these ingredients on melanogenesis were not evaluated.

#### 2.1.8. *Melia azedarach*

70% ethanol extract of *Melia azedarach* was demonstrated to rapidly increase melanin content in a concentration-dependent manner within 4 h [50]. Additionally, although the extract did not affect intracellular tyrosinase activity, protein levels of tyrosinase and TRP-2 at 2 and 4 h after treatment, it could improve TRP-1 protein expression at both time points.

#### 2.1.9. *Capparis spinosa* and *Erica multiflora*

Matsuyama and co-workers [51] also reported that extract of *Capparis spinosa* and *Erica multiflora* enhanced the synthesized melanin content in B16 cells without cytotoxity. Western blotting showed that tyrosinase expression was clearly increased in cells treated with the extracts as well.

#### 2.1.10. *Citrus paradisi*, *Citrus* *grandis*, *Fructus* *aurantii immaturus* and *Fructus* *aurantii*

Melanin content and tyrosinase expression in mouse B16 melanoma cells were assayed after treatment with four citrus plant extracts and their hydrolysates by Chiang et al. [52]. The results illustrated that hydrolysis increased the naringenin content in citrus extracts and that citrus preparations stimulated cellular melanogenesis and tyrosinase expression.

#### 2.1.11. Bee Venom

Jeon et al. [53] discovered that bee venom (BV) increased the number of human melanocytes dose and time dependently through PKA, ERK, and PI3K/Akt activation. The level of cAMP was also increased by BV treatment. Moreover, BV induced melanogenesis through increased tyrosinase expression and induced melanocyte dendricity and migration through PLA_2_ activation.

### 2.2. Active Natural Products/Synthesized Derivatives (Single Compounds)

#### 2.2.1. Flavonoids

##### Flavanones

In 2006, Ohguchi et al. [54] first examined the effect of naringenin (a naturallyoccurring citrus flavanone) on melanogenesis in mouse B16 melanoma cells. The study indicated that naringenin induced melanogenesis, and that the major melanogenic signaling factors, such as tyrosinase, Tyrp-1, Dct, and MITF, were upregulated by the compound.

**Figure 4:**
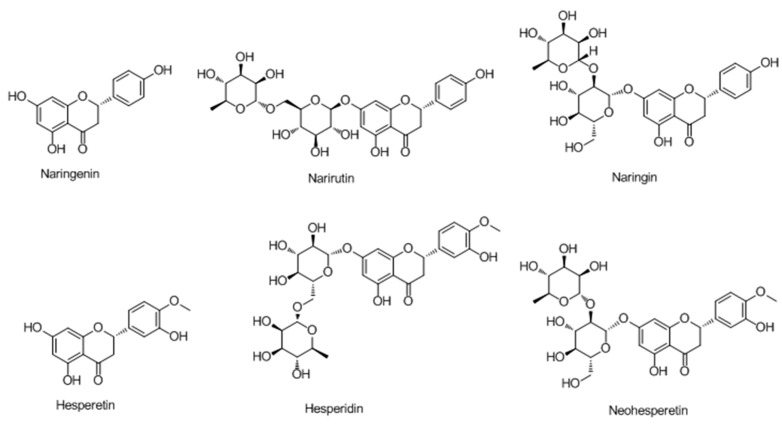
Structures of flavanones isolated from *Citrus*.

Huang et al. [55] reported that exposure of melanoma cells to naringenin (Figure 4) resulted in morphological changes accompanied by the induction of melanocyte differentiation-related markers, such as melanin synthesis, tyrosinase activity, and the expression of tyrosinase and MITF. They also observed an increase in the intracellular accumulation of β-catenin as well as the phosphorylation of glycogen synthase kinase-3β (GSK3β) protein after treatment with naringenin.

**Figure 5:**
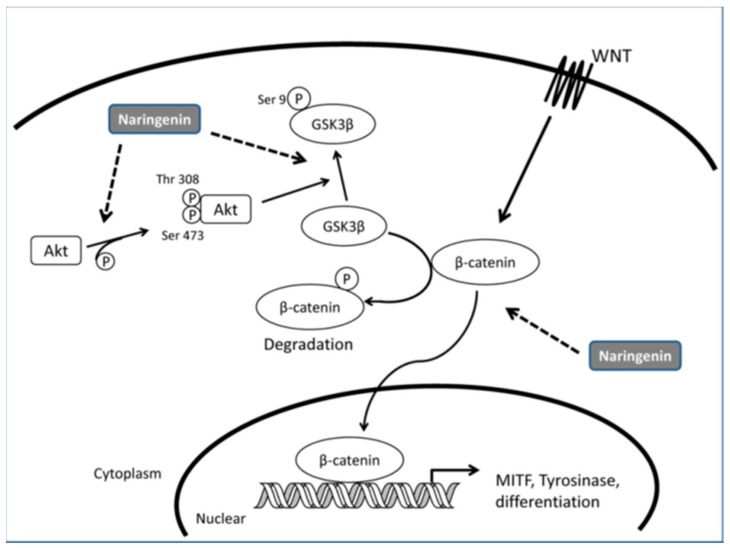
Model of signalling pathways involved in naringenin-induced melanogenesis [55].

Moreover, the activity of phosphatidyl-inositol 3-kinase (PI3K) was up-regulated by naringenin since the phosphorylated level of downstream Akt protein was enhanced. It was concluded that naringenin-induced melanogenesis through the Wnt-β-catenin-signalling pathway based on these results (Figure 5).

In their further study [56], it was found that the acid-hydrolyzed extracts of *Citrus sinensis*, *C. reticulata*, and *C. aurantium* enhanced melanin production. Hesperetin, which was the most abundant flavonoids in citrus hydrolyzed extracts and has a similar structure as naringenin, exhibited the best potency on melanin synthesis and induced tyrosinase and MITF expression. Moreover, it stimulated the activation of mitogen-activated protein kinases (MAPKs), phosphorylation of cAMP-responsive element binding protein (CREB) and glycogen synthase kinase-3β (GSK3β), and subsequently induced the accumulation of β-catenin.

**Scheme 2:**
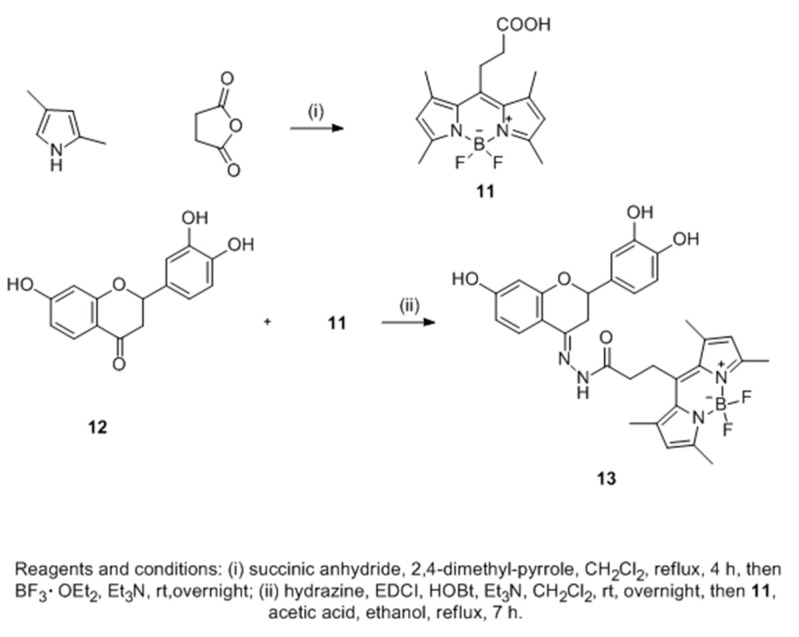
Synthesis of novel fluorescent flavanone-BODIPY probe **7** [57].

Sixteen 3′,4′,7-trihydroxyflavanones were synthesized to improve melanogenesis in B16-F10 mouse melanoma cell line (Scheme 2). In searching for their action target, a novel fluorescent flavanone-BODIPY probe without affecting its bioactivity was developed after structure-activity relationship (SAR) analysis of these compounds [57]. Confocal imaging showed that the probe which could increase the content of B16-F10 cells by selectively binding to its endoplasmic reticulum.

**Figure 6:**
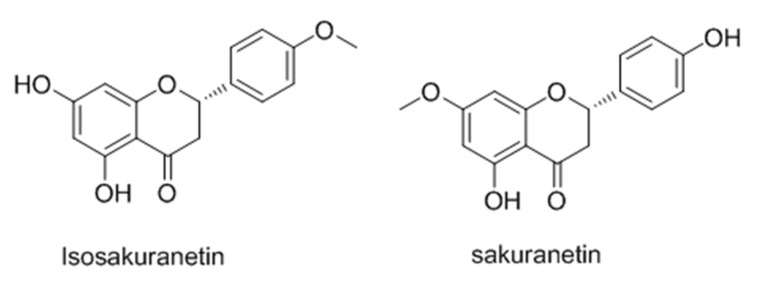
Structures of isosakuranetin and sakuranetin.

Isosakuranetin (Figure 6), an important component of propolis, *Baccharis dracunculifolia*, *Terminalia fagifolia*, *Citrus sinensis* [58,59,60], was proved to stimulates melanogenesis in B16 melanoma cells via up-regulation of MITF. Furthermore, it induced inhibition of ERK1/2 and PI3K/AKT signaling pathways activate MITF and subsequent expression of TYR, TRP1, and TRP2 [61].

##### Chalcones

Chalcone, which are one of the major classes of natural products with widespread distribution in fruits, vegetables, spices, tea and soy-based foodstuffs, display very versatile physiological activity [62,63,64,65]. Until now, most chalcones and their derivatives have been described as potent inhibitors of tyrosinase [66,67].

**Figure 7:**
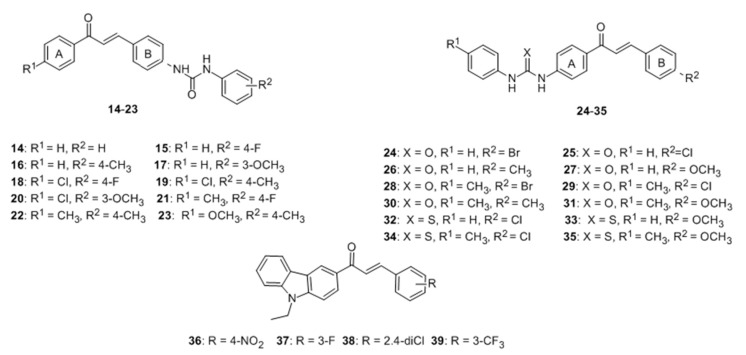
Structures of chalcone derivatives and analogues with activator effect on tyrosinase [68,69].

Few flavonoids have been reported as activators of tyrosinase. A new series of 4-(phenyl-urenyl)chalcones **14**–**23** and 4′-(phenylurenyl/thiourenyl)chalcone derivatives **24**–**35** were synthesized and their effects on the tyrosinase were evaluated. The results showed that **14**–**23** inhibited the PPO enzyme activity. Conversely, **24**–**35** exhibited activator effect on tyrosinase [68]. Nixha et al. [69] introduced a series of carbazole chalcone analogues and determined their activity on tyrosinase. From series, analogues **36**–**39** could enhance the activity of the enzyme. Unfortunately, these two series of compounds all showed poor activity (Figure 7).

**Figure 8:**
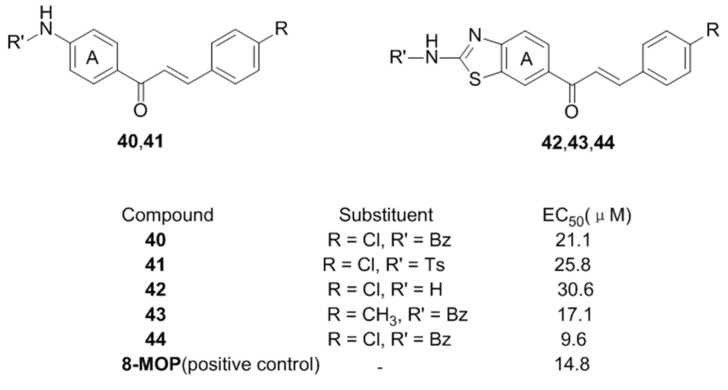
Structures of the benzothiazole and amide chalcones.

Inspired by above results, it was speculated that similar groups on the *para*-position of the chalcone A ring may cause a great improvement on activating effect on tyrosinase, but an inhibitor effect when it was introduced to B ring at the same position. Therefore, Niu et al. [70] first prepared sixteen chalcone derivatives containing benzothiazole and amide moieties and evaluated their activator effect on tyrosinase. Compared with the reference drug 8-MOP, compounds **40**, **41**, **42**, **43** and **44** displayed promising activity on tyrosinase with EC_50_ from 30.6–9.6 μM (Figure 8).

**Figure 9:**
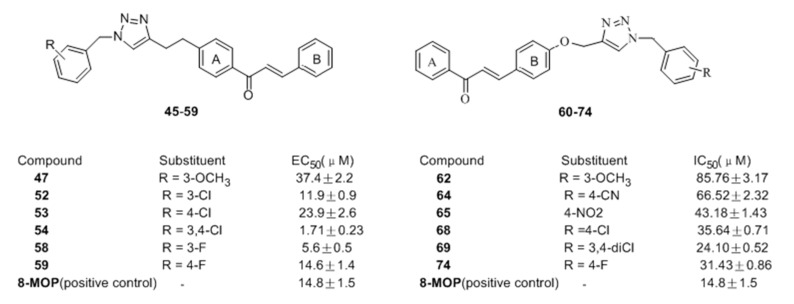
Structures of the chalcones derivatives bearing1,2,3-triazole moieties.

After that, substituted 1,2,3-triazoles were separately introduced into the A (compounds **45**–**59**) and B (compounds **60**–**74**) rings of the chalcone skeleton using click chemistry by our group [71]. The results showed that most of prepared compounds **45**–**59** have potent activating effect on tyrosinase, especially for **47**, **52**–**54** and **58**–**59**. Among them, compounds **54** and **58** demonstrated the best activity with EC_50_ =1.71 and 5.60 μM respectively, even better than the positive control (Figure 9).

On the contrary, compounds **62**, **64**–**65**, **68**–**69**, and **74** induced enzymatic inhibition on tyrosinase, which was consistent with our previous speculation.

**Figure 10:**
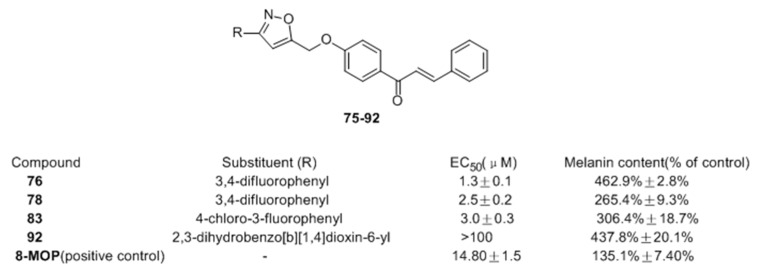
Structures of the chalcones derivatives bearing isoxazole moieties.

In 2016, Niu and co-workers [72] also reported the design and synthesis of novel chalcone derivatives **75**–**92** bearing isoxazole moieties as activators on tyrosinase and melanogenesis in murine B16 cells. Among the synthesized molecules, compounds **76**, **78**, and **83** exhibited the most potent activating effect on tyrosinase, with EC_50_ = 1.3, 2.5 and 3.0 μM, respectively. In B16 cells, it was interesting that derivatives substituted with halogens were generally more potent. Compounds **76** (463%) and **92** (438%) with 3 and 4-fold potency compared with 8-MOP (Figure 10), were recognized as the most promising candidate hits for further pharmacological study of anti-vitiligo.

**Figure 11:**
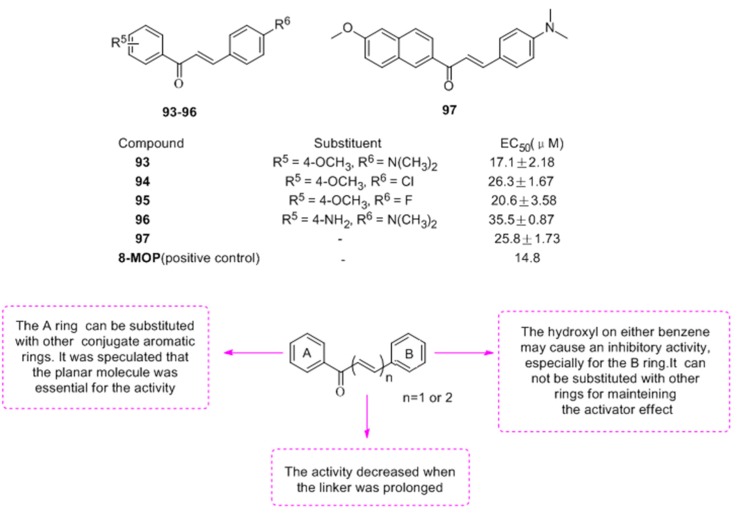
Structures of the chalcones with activator effect and SAR.

To find the pharmacophore of chalcone on tyrosinase, twenty-one chalcones and nine analogues were synthesized in view of three different components of chalcone (A, B ring and α-,β-unsaturated carbonyl) [73]. The biological evaluation revealed that most compounds (except polyhydroxy-chalcones) possess activator effects on tyrosinase, especially for **93**–**97** (Figure 11). Finally, compound **93** was found to increase melanin contents and tyrosinase activity 1.75- and 1.3-fold in B16 cells, respectively and the SAR of these tyrosinase activator was summed up for the first time as well.

##### Flavonoid glycosides

**Figure 12:**
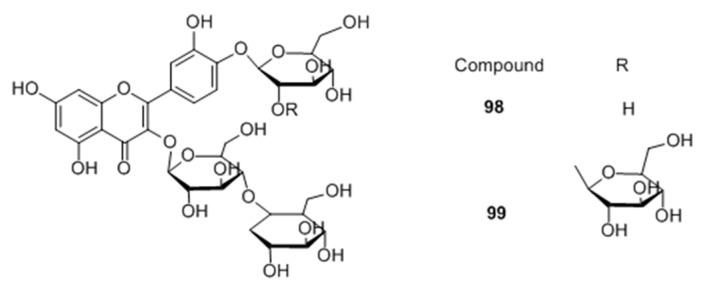
Structures of the compounds isolated from *Helminthostachys zeylanica.*

Two novel quercetin glucosides, were isolated from *Helminthostachys zeylanica* root 50% ethanol extract. Of the two quercetin-glucosides (Figure 12), compound **98** exhibited a high melanogenic acceleratory effect, 2.7 times higher than control at 10 μM concentration in murine B16 melanoma cells, with no cytotoxic effect [74].

**Figure 13:**
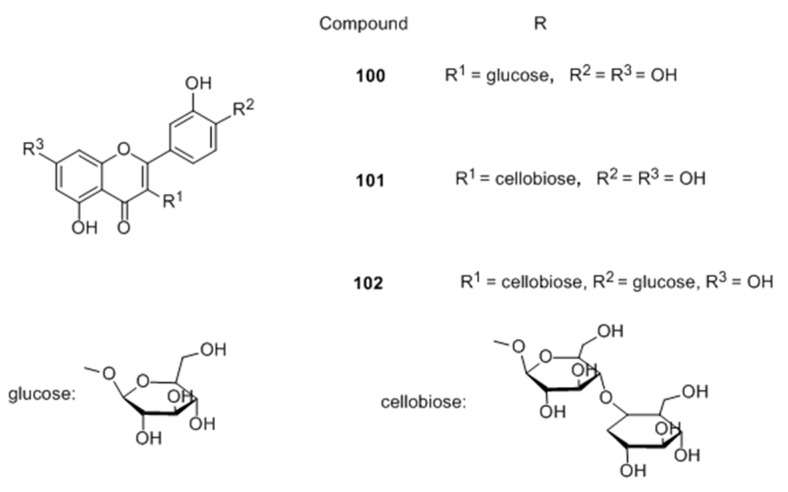
Structures of the synthesized quercetin glycosides.

In 2014, their group also derivatized a series of quercetin glycosides and evaluated them as melanogenesis acceleration compounds [75] (Figure 13), SAR was carried out to correlate the importance of many substituents with the observed activity. From the series, compound **100**, **101** and **102** showed more potent intracellular melanogenesis acceleration activities than theophyline used as positive control in a dose-dependent manner with no cytotoxic effect.

**Figure 14:**
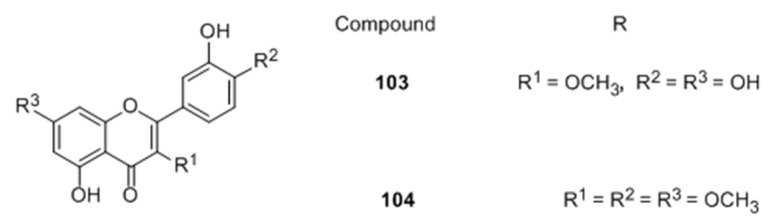
Structures of the synthesized quercetin derivatives.

Later, Yamauchi and co-workers [76] also attempted structure-guided synthesis of quercetin derivatives as melanogenesis activators based on their previous research. Among the prepared compounds, 3-*O*-methylquercetin (**103**) and 3′,4′,7-*O*-trimethylquercetin (**104**) increased melanin content more potently than the positive control with low cytotoxicity (Figure 14). However, the former increased the expression of tyrosinase and TRP-1 to a greater extent than the latter. Furthermore, compound **104** stimulated the expression of MITF and p-p38 MAPK as well, while they were not increased by **103**. These results suggested that **103** may enhance the expression of tyrosinase and TRP-1 by regulating the proteasomal degradation of melanogenic enzymes and/or by activating other transcriptional factors regulating enzyme expression.

**Figure 15:**
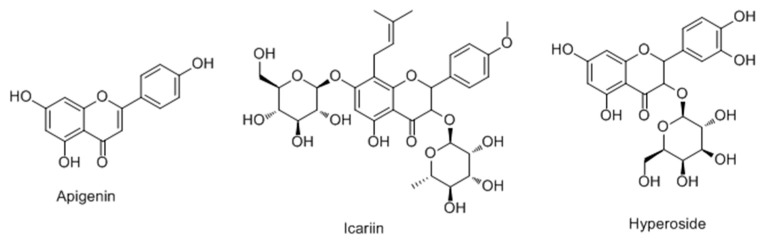
Structures of three compounds with activator effect on tyrosinase.

Ye et al. [77] first screened for tyrosinase activity enhancers among 35 phytocompounds in B16 mouse melanoma cells in 2010. Among them, three compounds (apigenin, icariin, hyperoside) were more potent than 8-MOP for enhancing tyrosinase activity and significantly increased cellular melanin contents without affecting cell proliferation (Figure 15). Western blot analysis demonstrated that these compounds could differentially increase the expression levels of tyrosinase, and TRP-1 and TRP-2. Together these data suggest that apigenin and icariin exert potent melanogenic activities through, at least in part, upregulating the protein expression levels of melanogenic enzymes in B16 cells.

##### Flavones

**Figure 16:**
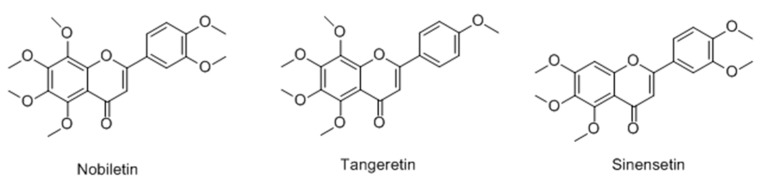
Structures of polymethoxylated flavones.

Yoon et al. [78] reported the stimulating effects of polymethoxylated flavones (nobiletin, tangeretin, sinensetin) on the production of melanin in murine B16-F10 melanoma cells (Figure 16). The results indicated all the tested compounds significantly increased melanin content, especially nobiletin. Further studies showed that nobiletin induced the expression of major melanogenic proteins such as tyrosinase, TRP-1 and that effect was probably related to the ERK–MAPK pathway.

**Figure 17:**
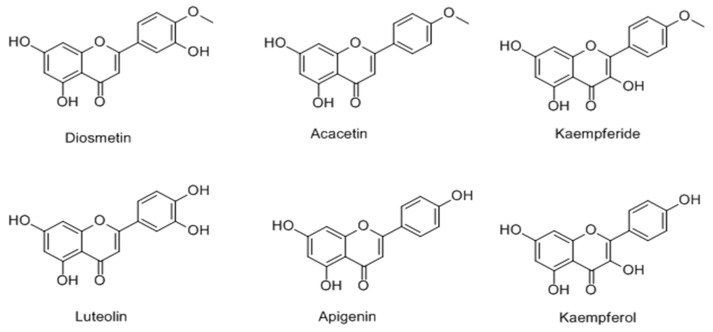
Structures of the4′-*O*-methylated and corresponding 4′-OH flavonoids.

In 2013, it was found that incubation of the B16-F10 cells with 10 μM 4′-*O*-methylated flavonoids (diosmetin, acacetin, kaempferide) (Figure 17), increased the melanin contents of the cells 3- to 7-fold higher than the control [79] and 20 μM acacetin exhibited the most promising activity with 33-fold higher activity than the vehicle. On the other hand, the corresponding 4′-OH-type flavonoids (luteolin, apigenin, kaempferol) possessed an obviously smaller effect. In addition, the upregulation of tyrosinase expression, preceded by activation of cAMP response element binding protein (CREB) and extracellular signal-regulated kinases types 1 and 2 (ERK1/2) was observed via melanogenic protein evaluation.

##### Isoflavones

**Figure 18:**
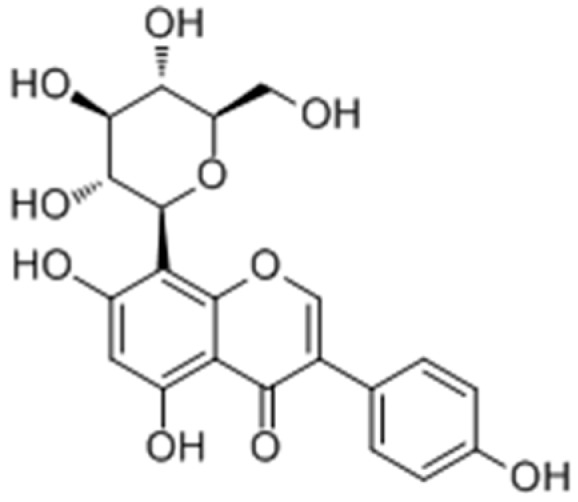
Structure of puerarin.

Park et al. [80] reported that the *Pueraria thunbergiana* (PT) extract and its major active ingredient puerarin (Figure 18) stimulated the melanogenesis via cAMP/MITF-M signaling pathway in vitro, and prevented the follicular depigmentation and vitiligo by stimulating melanin synthesis.

#### 2.2.2. Coumarins

For over a thousand years, the plant species *Ammi majus* L., *Psoralen corylifolia* L. and *Ficus carica* L. have often been used for repigmentation of skin with natural sunlight in India, Egypt and other oriental countries [81,82,83]. Similarly, their extracts were popular Uygur medicines used for vitiligo alone or in combination with XinJiang as well. Coumarins are widely distributed in these plants and have been isolated from their seeds, leaves and fruits [84,85,86].

Continuous studies have proved that these compounds show strong photosensitivity, which may be used for the treatment of vitiligo with subsequent exposure to long-wave ultraviolet radiation. Although the therapy was accompanied with some undesired side effects [87,88], it is still the most successful one for the disease today. Unfortunately, few coumarin derivatives possessing anti-vitiligo activity were reported.

**Figure 19:**
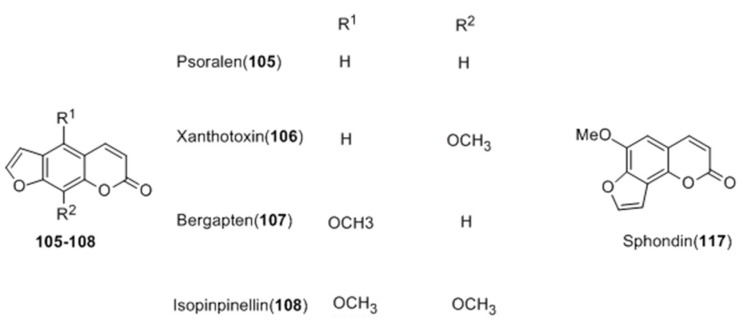
Structures of coumarins isolated from seven Umbelliferae crude drugs.

Early in 2005, seven ethanolic extracts from Umbelliferae crude drugs and sixteen coumarins (**105**–**120**) isolated were evaluated for their effects on melanin content using murine B16 melanoma cells [89]. Among them, the extract of *Heracleum lanatum*and and four compounds—psoralen (**105**), xanthotoxin (**106**), bergapten (**107**), isopimpinellin (**108**) and sphondin (**117**)—showed a potent stimulatory effect on melanogenesis. Moreover, the SAR was summarized according to the results (Figure 19).

**Figure 20:**
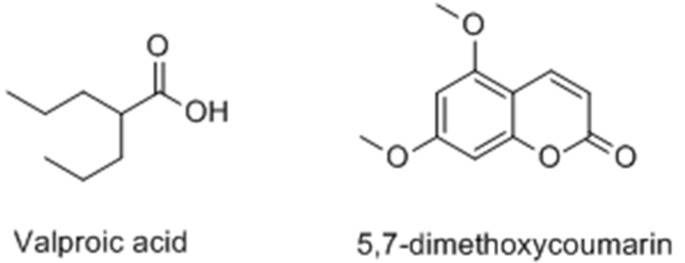
Structures of valproic acid and 5,7-dimethoxycoumarin.

Chodurek et al. [90] reported a new treament for malignant melanoma consisting of a combination of valproic acid (VPA) and 5,7-dimethoxycoumarin (DMC) (Figure 20). In A375 cells, the results demonstrated that both compunds could enhance the synthesis of melaninand the formation of dendrite and star-shaped cells. Moreover, upregulation of tyrosinase activity and gene expression were observed in response to VPA treatment.

**Figure 21:**
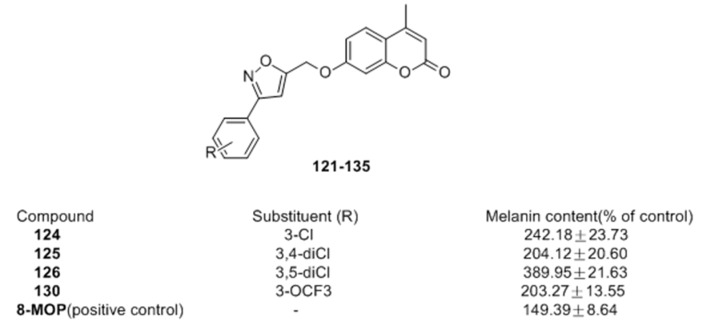
Structures of the coumarin derivatives bearing isoxazole moieties.

Recently, Pang et al. [91] of our group prepared coumarin derivatives bearing isoxazole moieties (**121**–**135**) as melanogenic stimulator from suitable 5-(bromomethyl)isoxazoles and 4-methylumbelliferone. Among the synthesized molecules, compounds **124** and **126** exhibited excellent potency on melanin synthesis with nearly 1.6- and 2.6-fold potency compared with 8-MOP (149%) respectively, in murine B16 cells (Figure 21).

**Figure 22:**
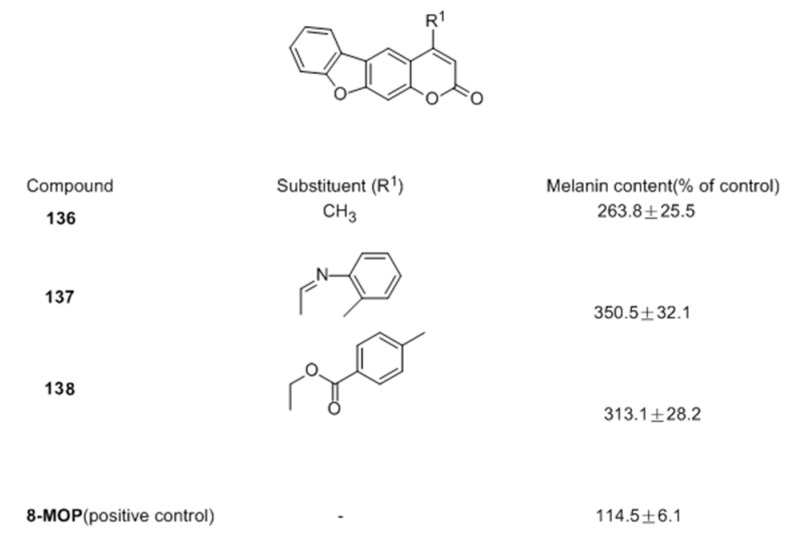
Structures of the synthesized furocoumarin derivatives.

In another study, our researchers also reported the synthesis of twenty-five furocoumarin derivatives and evaluated the stimulatory effect of them on melanogenesis in murine B16 cells [92]. In this series, twenty-three compounds were more potent than the positive control (8-MOP). Compounds **137** (350.5%) and **138** (313.1%) based on the scaffold of **136** were nearly 3-fold stronger than 8-MOP (Figure 22).

#### 2.2.3. Terpenoids

**Figure 23:**
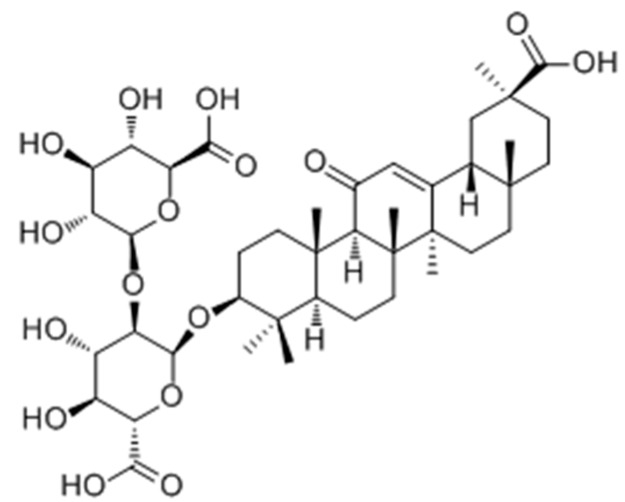
Structure of glycyrrhizin isolated from *Glycyrrhiza glabra* L.

Following the report of stimulation of melanogenesis by glycyrrhizin (GR, Figure 23) via increasing tyrosinase expression at mRNA and protein levels, Lee et al. [93] studied the molecular mechanism of the process. The results indicated that GR induces melanogenesis by elevating intracellular cAMP level. In addition, it was able to activate both AP-1 and CRE pathways by increasing intracellular cAMP level.

**Figure 24:**
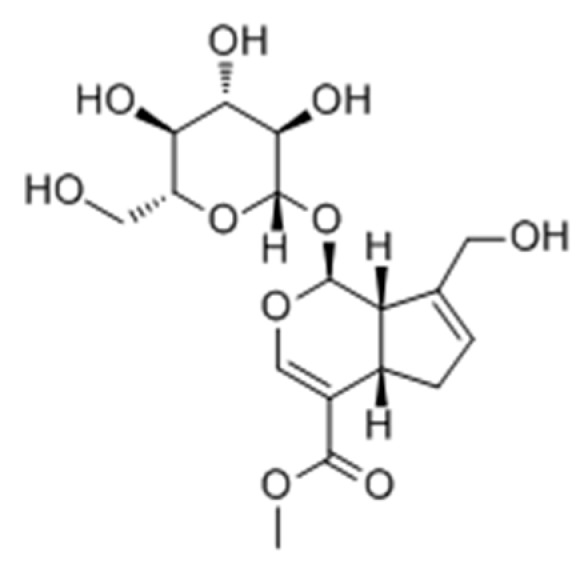
Structure of geniposide isolated from *Gardenia jasminoides Ellis* fruits.

Geniposide (Figure 24) isolated from the fruit of *Gardenia* *jasminoides* *Ellis* was used as a Chinese traditional medicine for treatment of generalized vitiligo. In 2008, Lan et al. [94] studied the action and mechanism of geniposide’s enhancement of melanogenesis in norepinephrine-exposed normal human epidermal melanocytes. From the results, it was suggested that geniposide can enhance melanogenesis by stem cell factor/c-kit signalling in which the expression of c-kit receptor is augmented in norepinephrine-exposed normal human epidermal melanocyte.

**Figure 25:**
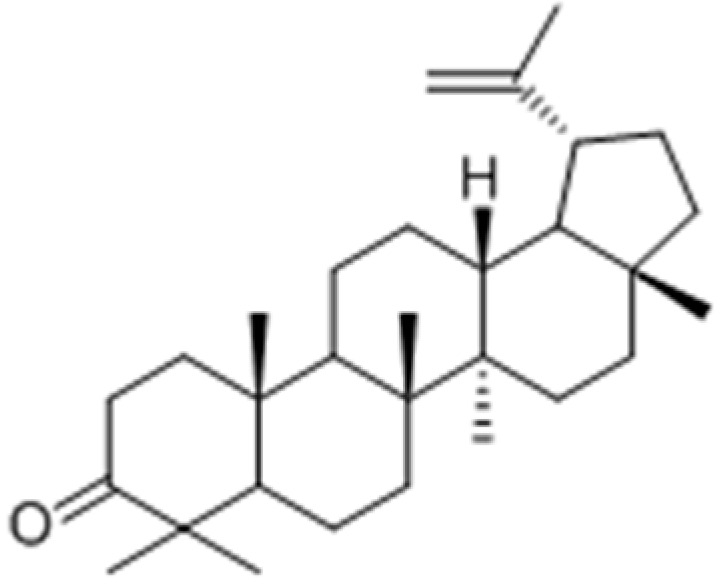
Structure of lupenone isolated from leaf effects extracts of *Erica* *multiflora*.

Villareal and co-workers [95] evaluated the melanogenesis stimulatory effects of leaf extracts of *Erica* *multiflora* and its active component lupenone (Figure 25) as possible therapeutic agents to address hypopigmentation disorders. The results showed that *E. multiflora* ethyl-acetate extract and lupenone enhanced melanogenesis by increasing the tyrosinase enzyme expression via mitogen-activated protein kinase phosphorylated extracellular signal-regulated kinases 1 and 2 phosphorylation inhibition.

**Figure 26:**
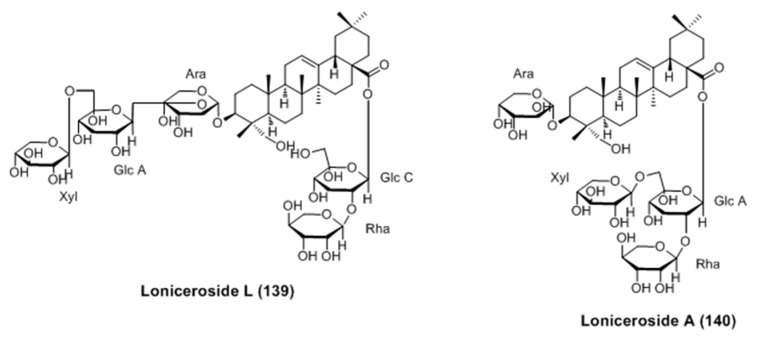
Structure of triterpene glycosides isolated from aerial parts of *Weigela subsessilis*.

Three new triterpene glycosides (lonicerosides K, L and M) and eleven known compounds were isolated from the aerial parts of *Weigela subsessilis* [96]. Among them, lonicerosides A (**140**) and L (**139**) were proved to stimulate melanogenesis without cytotoxicity in murine B16-F0 melanoma cells (Figure 26). Furthermore, the expression of tyrosinase and MITF proteins were upregulated by both of them according to western blot analysis.

**Figure 27:**
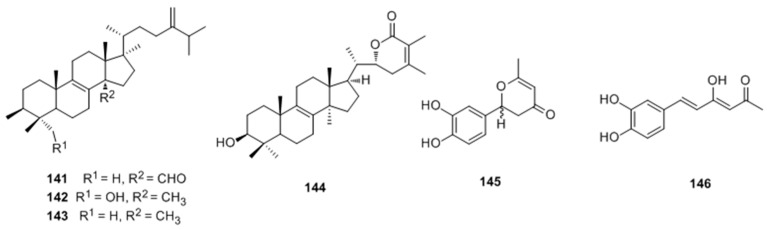
Structure of triterpenoids and phenolic compound isolated from fruiting bodies of *Inonotus hispidus*.

Ren et al. [97] reported the isolaton and structure identification of two new triterpenoids (hispindic acids A (**141**) and B (**142**) and a new phenolic compound hispinine (**145**), along with nine known compounds, from the fruiting bodies of *Inonotus hispidus*. Isolates **141**–**146** were found to exhibited stronger activate abilities of melanogenesis and tyrosinase in B16 melanoma cells than those of positive control (8-MOP) at 50 μmol/L (Figure 27).

#### 2.2.4. Resveratrols

**Figure 28:**
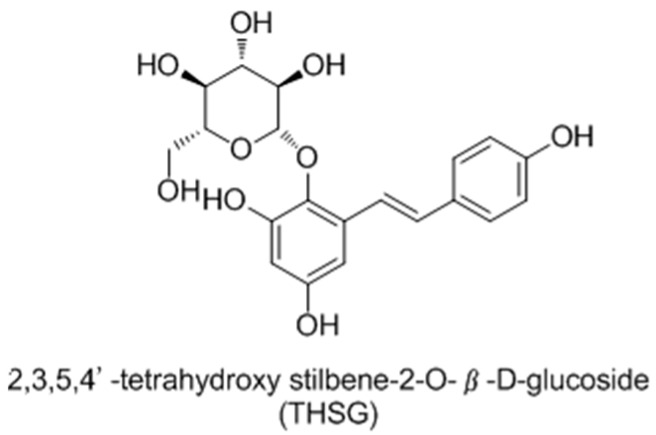
Structure of THSG isolated from dried tuber root of *Polygonum multiflorum*.

Early in 2008, Guan and co-workers [98] reported 2,3,5,4′-tetrahydroxystilbene-2-*O*-β-d-glucoside (THSG) (Figure 28) as tyrosinase activator andmelanogenesisstimulator without cytotoxicity in B16 melanoma cells. In further studies, it was found to induce melanin production via increasing the mRNA and protein levels of tyrosinase. Western blotanalysis revealed that THSG exerts its stimulatory effect on melanogenesis by MAP kinase activation and MITF induction of tyrosinase [99].

**Figure 29:**
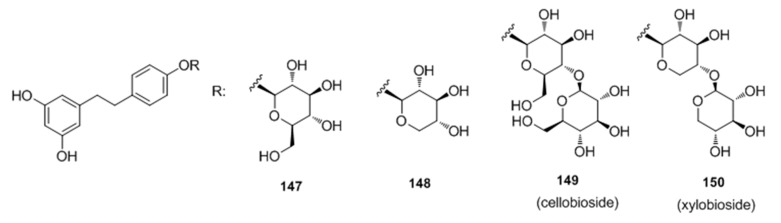
Structures of dihydroresveratrol glucosides.

Recently, Oode et al. [100] described a facile synthesis of cellobioside (**149**) and xylobioside (**150**) based on naturally occurring melanogenesis-controlling agent dihydroresveratrol glucoside via Schmidt glycosylation (Figure 29). Both analogues **149** and **150** stimulated melanogenesis with efficacies comparable to that of 8-MOP, which suggested that diglycosyl modification of the 4′-OH on the dihydroresveratrol skeleton leads to the activation of melanogenesis, both with and without hydroxymethyl groups in the sugar moieties.

#### 2.2.5. Aurones

**Figure 30:**
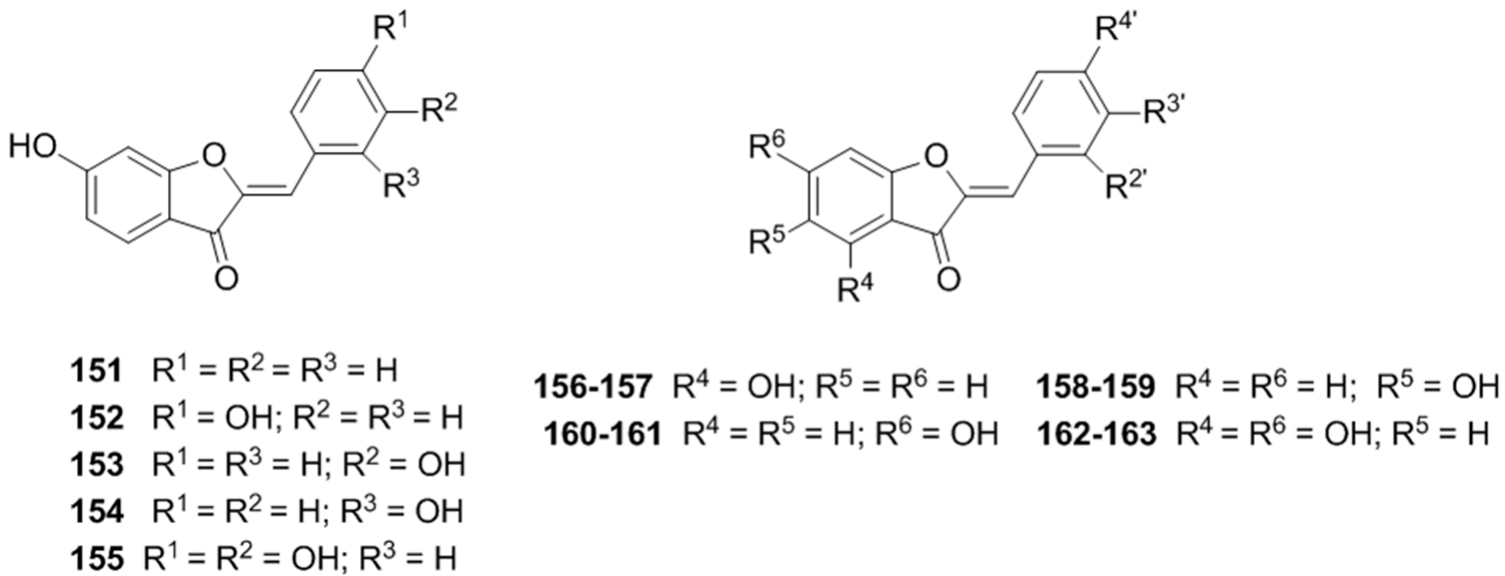
Structure of the synthetic aurones.

In 2012, Dubois et al. [101] synthesized several aurones **151**–**155** and discovered that **153** and **154** behaved as hyperbolic activators of mushroom tyrosinase; Later, aseries of twenty-four aurones with different hydroxylation patterns on A, B rings were prepared and evaluated for their abilities on tyrosinase from mushroom to bacterial respectively by the same group [102], the results showed that **156**–**157**, **158**–**159**, **160**–**161** and **162**–**163** can improve the activity of mushroom tyrosinase (Figure 30).

#### 2.2.6. Polyphenols

**Figure 31:**
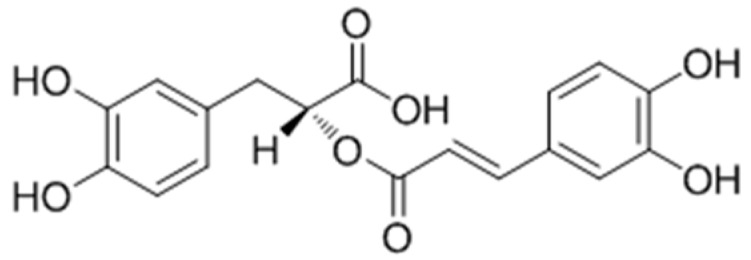
Structure of rosmarinic acid.

It has been reported that the extracts of *Salvia officinalis* L. increased the melanin production without necessarily changing the enzymatic activity in B16-F10 cells. Moreover, rosmarinic acid (Figure 31), the main phenol derivative isolated from the extracts was found to exhibit a dual behavior on melanogenesis, increasing melanin biosynthesis and tyrosinase activity at low concentrations and decreasing them at higher levels [103]. Based on these findings, Lee et al. [104] studied the molecular events of pigmenting effect of rosmarinic acid in B16 melanoma cells. After several experiments, it was believed that the compound induced melanogenesis through the PKA activation signaling.

#### 2.2.7. Other Compounds

**Figure 32:**
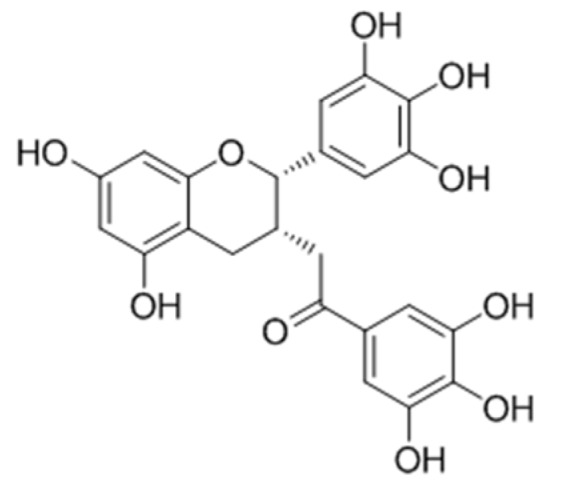
Structure of epigallocatechin-3-gallate.

In 2014, Zhu et al. [105] assessed the role of epigallocatechin-3-gallate (EGCG) (Figure 32) in vitiligo induced by monobenzone in mice. It was demonstrated that EGCG could delay the time of depigmentation, reduce the prevalence of depigmentation and decrease the area of depigmentation by reducing excessive inflammatory responses, especially infiltration of CD8+ T cells and inhibiting the levels of inflammatory mediators. In addition, gene-expression profile of the model in relation to EGCG was studied as well. A total of 1264 down-regulated genes and 1332 up-regulated genes were recorded in the 5% EGCG group compared with the model group using whole genome oligo microarray assay. Thesere sults suggested that EGCG may significantly decrease the risk of vitiligo.

**Figure 33:**
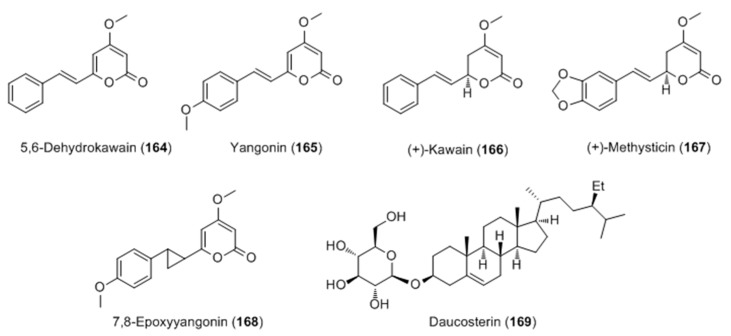
Structure of kavalactones and glucosylsterols isolated from the rhizome extract of *Piper methysticum*.

The extract of *Piper methysticum* rhizome (Kava) was identified as the most potent agent on melanogenesis in B16 cells among different parts of five *Piper* species [106]. Activity-guided fractionation of Kava extract led to the isolation of six compounds, with two active kavalactones, yangonin (**165**) and 7,8-epoxyyangonin (**168**), a glucosyl-steroldaucosterin (**169**), which all possess a significant stimulatory effect on melanogenesis (Figure 33).

**Figure 34:**
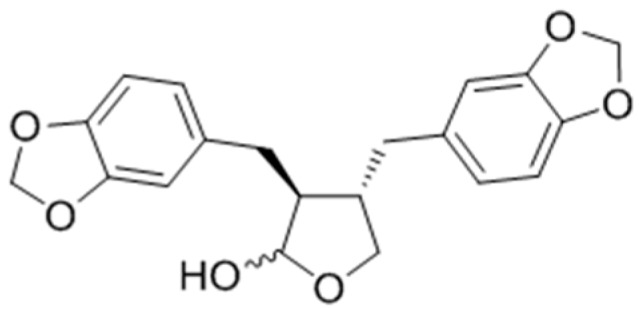
Structure of (−)-cubebin.

**Figure 35:**
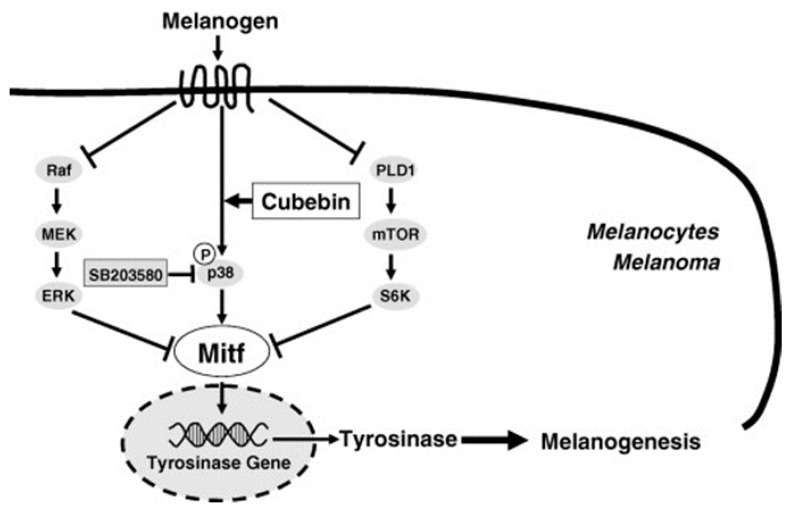
A proposed scheme showing the activation mechanism of cubebin on melanogenesis [107].

Hirata and co-workers had discovered that the natural product (−)-cubebin (Figure 34) isolated from leaves of *Piper nigrum* L. wa sproved to have a stimulator effect on melanogenesis and tyrosinase activity in murine B16 melanoma cells without any significant effects on cell proliferation [107]. The expression levels of tyrosinase and tyrosinase mRNA were both enhanced after addition of cubebin. Western blot analysis revealed that melanogenesis induced by cubebin was attributable to the increase of tyrosinasegene expression through positive regulator, MITF, initiated by cubebin-induced activation of p38-MAPK, as shown in Figure 35.

**Figure 36:**
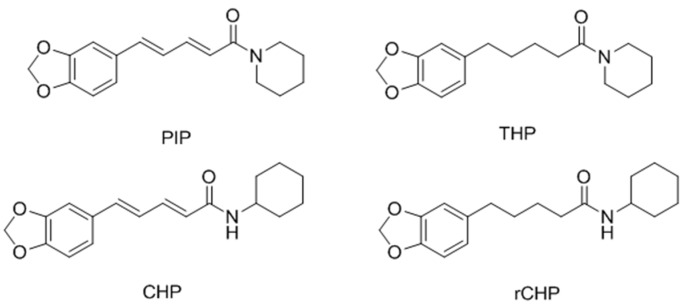
Structure of PIP and three analogues.

In 2008, Faas et al. [108] investigated the ability of PIP and three analogues (THP, CHP and rCHP, Figure 36) to stimulate pigmentation in a strain of sparsely pigmented mice. The results showed that treatment with PIP, THP or rCHP and UVR induced a marked pigmentation response in HRA/Skh-II mice, with clinically better results than UVR alone, which supported the potential use of these compounds in treating vitiligo.

## 3. Conclusions

Melanogenesis stimulators are important as skin-pigmentation agents in several dermatological problems, such as vitiligo. Over the last ten years, a number of melanogenesis stimulators have been reported from natural and synthetic sources. Although none of them have revealed the precise therapeutic target of the vitiligo, many possible mechanisms of these stimulators in melanogenesis were proposed to help us understand this complicated disease.

Many melanogenic stimulating agents target catalytic activity of TYR. TYR catalyzes the process of neuromelanin production, in which oxidation of dopamine produces dopaquinones. However, excessive production of dopaquinones results in neuronal damage and cell death [109]. This links TYR to several neurodegenerative disorders such as Parkinson’s as mentioned above [110]. In addition to that, other approaches to melanogenesis stimulators include an increase and activation of the expression of melanogenesis-related proteins such as MITF, TYR, TRP-1 and TRP-2, resulting from the modulation of various signaling pathways.

**Figure 37:**
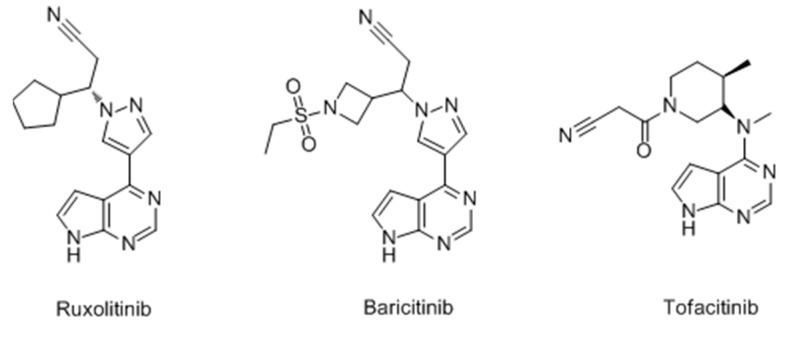
Structure of JAK inhibitors.

However, it is a pity that most of these melanogenesis stimulators are still in the drug discovery phase, and few pharmacologic actions and adverse reactions in vivo are reported as a result of the difficulty in building the related animal models. According to the current research progress and problems, it was more efficient to develop new drugs from traditional Chinese medicines, such as Uighur medicine. Many drugs for vitiligo have already been available on the market for years. Qubaibabuqi and Kaliziri [46,111,112], which exhibit attractive therapeutic effects clinically with few side effects, were popular in Xinjiang and its neighboring central Asian countries. Besides, JAK inhibitors (Figure 37), often used for myelodysplastic disorders, were proven to be efficacious for vitiligo recently and a large number of clinical trials are currently underway [113,114]. JAK inhibitors are likely to have broad applicability in dermatology and more lead compounds for vitiligo may be explored among these inhibitors in view of their clear target.

In conclusion, we hope that this perspective will be useful to medicinal chemists working on melanogenesis and related proteins to identify novel melanogenesis stimulators with drug-like properties.

## Figures and Tables

**Table 1:** Summary of stimulation effect on tyrosinase, melanogenesis and other factors in cultured cells and pigmentation responsein animal of different plants.

Botanical Name	Extraction Parts	Solvents	Items Up-Regulated	Types of Cultured Cells	Related Signaling Pathway	Animal Experiments	Ref.
*Daphne gnidium*	Leaf	Ethyl acetate	Melanin, Tyrosinase activity	B16-F0	Ns *^a^*	Ns	[37]
*Daphne gnidium*	Leaf	Chloroform	Melanin, Tyrosinase activity	B16-F0	Ns	Ns	[38]
*Moricandia arvensis*	Leaf	Chloroform	Melanin, Tyrosinase activity	B16-F0	Ns	Ns	[39]
*Polygonum multiflorum*	Root	Water	MITF, Melanocyte migration	Human melanocytes	Ns	Ns	[40]
*Eclipta Prostrate*	Whole herb	Water	Melanin, Tyrosinase activity, MITF, Melanocyte migration	Human melanocytes	Ns	Ns	[40]
*Rehmannia Glutinosa*	Root	Water	Melanin, MITF	Human melanocytes	Ns	Ns	[40]
*Cassia alata*	Leaf	Nm *^b^*	Melanin, Tyrosinase activity, Dendritogenesis, Migration	Melb-a melanoblast	Ns	Ns	[41]
*Cassia occidentalis*	Leaf	Methanol	Melanin, Tyrosinase activity, Dendritogenesis, Migration	Melb-a melanoblast	Ns	Ns	[42]
*Pyrostegia venusta*	Leaf, Flower	Ethanol:Water (70:30, *v/v*)	Melanin	B16-F10	Ns	Ns	[43]
*Pyrostegia venusta*	Leaf	Ethanol:Water (70:30, *v/v*)	Epidermal melanin, Dermal pigmentation	-	Ns	C56BL/6 mice	[44]
*Vernonia anthelmintica*	Fruit	Ethanol:Water (60:40, *v/v*)	Melanin, Tyrosinase activity, Tyrosinase expression, p38 MAPK phosphorylation, MITF expression	B16-F10, Human melanocytes	p38 MAPK	Ns	[45]
*Vernonia anthelmintica*	Seed	Ethanol:Water (80:20, *v/v*)	TYR, TRP-1, TRP-2 and MITF expression	B16 murinemelanoma	Ns	Ns	[46]
*Melissa officinalis*	Whole herb	Nm	Melanin	Human keratinocytes	Ns	Ns	[49]
*Melia azedarach*	Nm	Ethanol:Water (70:30, *v/v*)	Melanin, TRP-1 expression	B16-F10	Ns	NS	[50]
*Capparis spinosa*	Nm	Nm	Melanin, Tyrosinase expression	B16 murinemelanoma	Ns	Ns	[51]
*Erica multiflora*	Nm	Nm	Melanin, Tyrosinase expression	B16 murine melanoma	Ns	Ns	[51]
*Citrus paradisi*, *Citrus grandis*, *Fructus* *aurantii* *immaturus*, *Fructus* *aurantii*	Rind	Ethyl acetate	Melanin, Tyrosinase expression	B16 murine melanoma	Ns	Ns	[52]

*^a^* Ns means not studied; *^b^* Nm means not mentioned.

## Abbreviations

UV	Ultra-violet
α-MSH	α-Melanocyte-stimulating hormone
SCF	Stem cell factor
ET-1	Endothelin-1
NO	Nitric oxide
ACTH	Adrenocorticotropic hormone
MITF	Microphthalmia-associated transcription factor
TYR	Tyrosinase
TRP-1	Tyrosinase-related protein 1
TRP-2	Tyrosinase-related protein 2
Dct	Dopachrome tautomerase
L-DOPA	3,4-Dihydroxyphenylalanine
DQ	Dopaquinone
DHI	5,6-Dihydroxyindole
DHICA	5,6-Dihydroxyindole-2-carboxylic acid
IQ	Indole-5,6-quinone
5-S-CD	5-S-cysteinyldopa
2-S-CD	2-S-cysteinyldopa
p38 MAPK	p38 Mitogen-activated protein kinase
8-MOP	8-Methoxypsoralen
PMRP	Polygoni multiflori radix praeparata
EH	Ecliptae herba
RRP	Rehmanniae radix praeparata
TNF-α	Tumor necrosis factor
CGA	Chlorogenic acid
RA	Rosmarinic acid
BV	Bee venom
GSK3β	Glycogen synthase kinase-3β
PI3K	Phosphatidylinositol 3-kinase
MAPK**s**	Mitogen-activated protein kinases
EDCI	1-Ethyl-3-(3-dimethylaminopropyl)carbodiimide hydrochloride
HOBt	1-Hydroxybenzotriazole
SAR	Structure-activity relationship
CREB	cAMP response element binding protein
ERK	Extracellular signal-regulated kinases
AP-1	Activator protein-1
PKA	Protein kinase AA
